# Coupling of electrochemical–temperature–mechanical processes in marine clay during electro-osmotic consolidation

**DOI:** 10.1038/s41598-020-70700-z

**Published:** 2020-08-18

**Authors:** Zhi-Jia Xue, Chang-Gen Yan, Wu-Gang Li

**Affiliations:** 1 School of Highway, Chang’an University, Xi’an, 710064 Shaanxi China; 2 State Key Laboratory of Coastal and Offshore Engineering, Dalian University of Technology, Dalian, 116024 Liaoning China; 3 School of Environment and Civil Engineering, Jiangnan University, Wuxi, 214122 Jiangsu China

**Keywords:** Civil engineering, Electrical and electronic engineering

## Abstract

Electro-osmotic consolidation has been applied in several geotechnical engineering applications that contain a series of complex processes, including electrochemical processes, temperature changes, and mechanical evolution. To explore the combination of electrochemical–temperature–mechanical processes in marine clay, electro-osmotic consolidation experiments were conducted using a self-made electro-osmotic consolidation system under various durations and voltages. The following findings was obtained: (1) the change in the pH value increased during electro-osmotic consolidation and as the voltage rise; (2) the temperature increased with a rise in voltage in the initial stage of the experiments, which was induced by Joule heating; (3) the temperature rise promoted the electro-osmotic consolidation process, which included a rise in the coefficient of consolidation and a reduction in water content; (4) horizontal shrinkage occurred when the horizontal stress increment was greater than the critical stress condition. In addition, the volume difference reached a constant value, and was proportional to the voltage rise. After the discussion, a coupling analysis was conducted, which can help to better understand the mechanism of electro-osmotic consolidation and can provide reference for engineering applications.

## Introduction

Civil engineers attempt to use the offshore reclamation method to satisfy the development requirements of coastal cities. For example, Australia (Brisbane, Queensland)^1^, China (Dalian, Jinzhou Bay)^2^, and Singapore^3^ carried out coastal reclamation engineering for city planning purposes. Surcharge preloading and vacuum preloading can both be applied successfully in the area of reclamation. However, these two methods face a long construction period in the development of soft clay because the permeability coefficient of clay is low, which is in the range of 10^–8^ m/s^4^. In comparison, electro-osmotic consolidation is independent of the size of soil particles^5^. Direct current causes pore water to move from the anode to the cathode^6^. Therefore, electro-osmotic consolidation is an effective method to improve the foundation, slope, and subgrade of soft clay. Electro-osmotic consolidation is a complex process, involving electrochemical processes, temperature changes, and mechanical evolution^5^.

An electrochemical process is a particularly important process during electro-osmotic consolidation. It is responsible for the electrolysis reaction and the transport of ions. Liaki et al.^8^ measured the pH value of clay after different electro-osmotic consolidation treatment durations using a stainless steel anode. They found that the pH value steadily decreased near the anode and increased near the cathode. Xue et al.^9^ observed that the pH value of clay near a copper anode was higher than that of a stainless steel anode, which was mainly because the product of chlorine consumed the electric charge and there was not much electric charge on the product of H^+^ in copper anode condition. Wu et al.^10^ measured the pH value after electro-osmotic consolidation experiments on bentonite clay. The pH value decreased to ~ 3.5 near the anode and increased to ~ 8.8 near the cathode.

Burnotte et al.^11^ monitored the temperature of the foundation of soil during an electro-osmotic field test in Canada, and they found that the maximum temperature was 91 °C near the anode. Chen et al.^12^ observed that the maximum temperature was 42 °C in the electro-osmotic consolidation field test. Therefore, a temperature rise was very obvious in the above two kinds of soils during electro-osmotic consolidation experiments.

During electro-osmotic consolidation, vertical settlement of the soil occurred due to the generation of negative pore water pressure^13^. Peng et al.^14^ monitored the settlement near the anode and the cathode, and found that the settlement of the anode was much higher than that of the cathode. Estabragh et al.^15^ found that the rate of settlement increased with a rise in the electric potential gradient value.

Jeyakanthan et al.^16^ observed the horizontal shrinkage phenomenon under isotropic conditions, which included the contribution of the horizontal stress of the hydraulic confining pressure. Xue et al.^13^ also observed the horizontal shrinkage of marine clay samples after electro-osmotic consolidation treatment with under *k*_*0*_ consolidation conditions. However, they did not analyze the horizontal shrinkage development process. As known from previous studies, the drainage volume increases^11,17–19^ and the water content of clay reduces during electro-osmotic consolidation^20–22^. Due to the occurrence of horizontal shrinkage, the drainage volume is higher than the vertical volume change (vertical volume change = vertical settlement × area of foundation). However, seldom does the literature focus on this phenomenon.

In addition, it is also important to analyze the combination of electrochemical–temperature–mechanical processes during the electro-osmotic consolidation of marine clay. Therefore, this paper conducted a series of electro-osmotic consolidation experiments under three durations (12, 24, and 48 h) and three voltages (6, 9, and 12 V) under *k*_*0*_ consolidation conditions (i.e., limit the outward lateral deformation of soil). During the electro-osmotic consolidation experiments, the current, temperature, settlement, and drainage volume were monitored. After the electro-osmotic consolidation experiments, the horizontal shrinkage, water content, pH value, and molar concentration of chloridion were measured. Based on the electro-osmotic consolidation theory, the effective stress principle, and the transport of ion theory, this paper provided an explanation of the combination of electrochemical–temperature–mechanical evolutive processes.

## Materials and methods

### Description of clay

The clay sample was collected from Dalian Dayao Bay, which was deposited in an offshore environment. The mineralogy of the clay is illite 65%, smectite 14% and kaolinite 12%, which was measured by X-ray diffraction (XRD) following the method in previous literature^23^. The air-dried clay sample was pulverized by a mallet, and then sieved with a 2 mm sieve. The pore composition fluid was evaluated by a similiar method described in the previous literature^24^. Next, slurry sample (clay mass/distilled water mass = 1:5) were made, and then the supernatant solution was obtained by a centrifugal machine (CT15RT). The molar concentrations of the main cations of solute salt were as follows: c(Na^+^) = 0.041 mol/L, c(Ca^2+^) = 0.001 mol/L, c(Cl^−^) = 0.046 mol/l, which were measured by an ionometer (PXSJ-216F). The initial pH value was 7.17, which was measured by a pH meter (METTLER TOLEDO FE-28).

**Figure 1:**
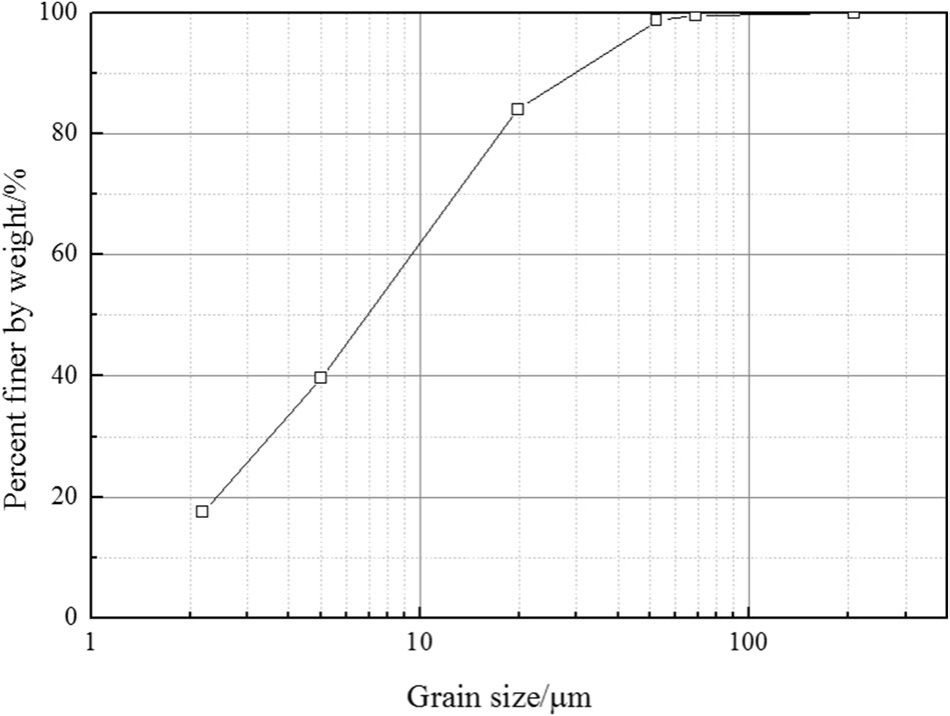
Grain size distribution of the marine clay.

Based on the soil test standard SL237-1997, the liquid and plastic limits were measured, and the values are shown in Table 1. The liquid limit was less than 50% and the plasticity index was 21%. The clay sample belonged to CL, according to the Unified Soil Classification System (USCS). Next, a slurry sample was made with 60% water content, which was much higher than the liquid limit and was in the flowing plastic state. The salt content was 1.35% based on the soil test standard SL237-1997. We used ultrasonic dispersion method to make the suspension, and then measured the grain size distribution with a laser particle analyzer (Malvern AWM2000) following the method in previous literature^25^. Figure 1 shows the grain size distribution: the < 0.005 mm fraction corresponded to 39.6%. After the end of the 25 kPa primary consolidation process, this paper obtained a consolidation water content of 38.8%.

**Table 1:** Properties of the marine clay samples.

Properties	Value
Water content (%)	60
Liquid limit (%)	42.7
Plastic limit (%)	21.7
Salt content (%)	1.35
pH Consolidation water content (%)	7.17 38.8
**Mineralogy of the clay (%)**	
Illite	65
Smectite	14
Kaolinite	12

### Experimental apparatus

**Figure 2:**
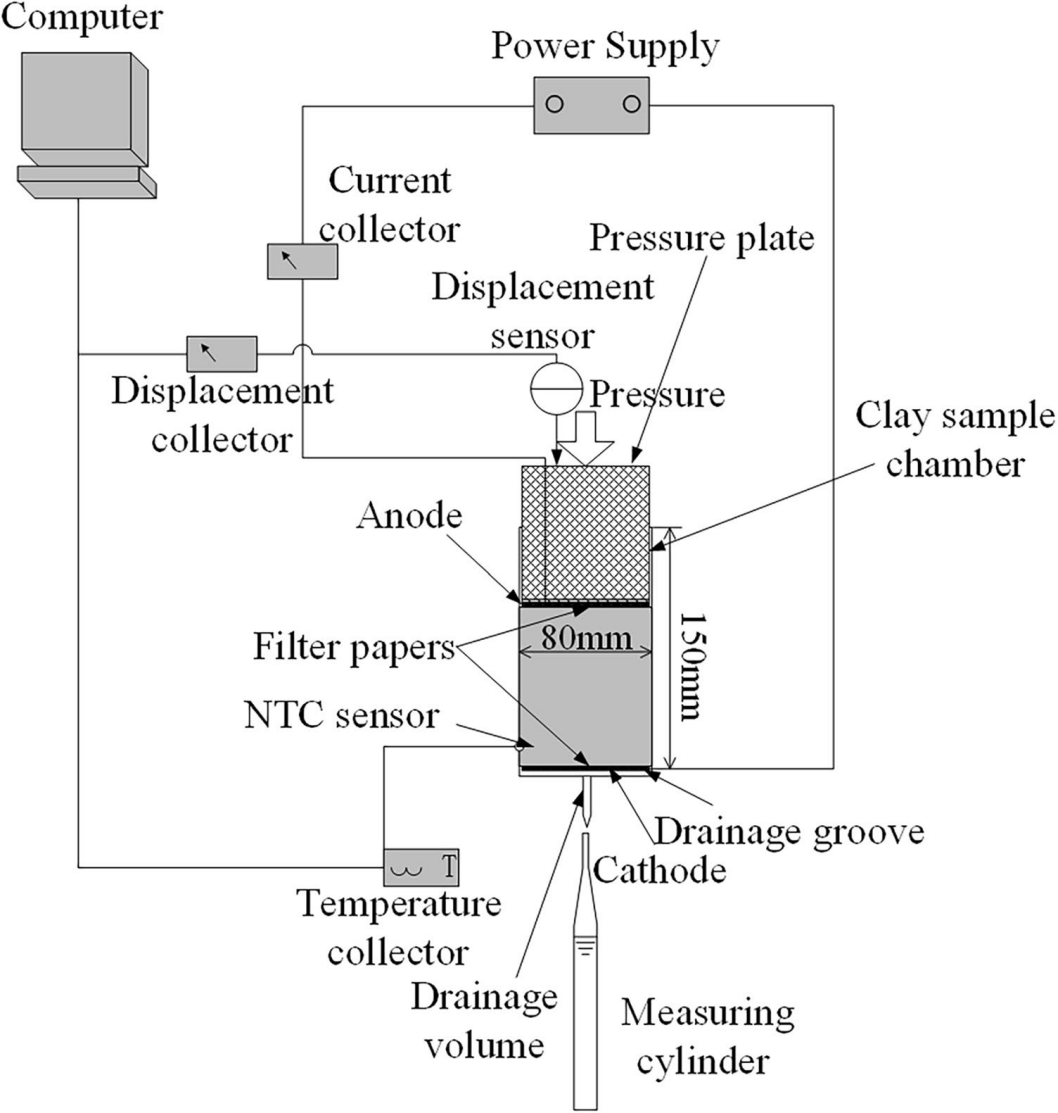
A diagram of the apparatus system^9^.

To satisfy the research requirements, an electro-osmotic consolidation experiment system was designed and made in this study, including an electro-osmotic consolidation box, a power supply system, and a data monitoring system. A diagram of the apparatus system is shown in Fig. 2.

The electro-osmotic consolidation box included a clay sample chamber and a pressure loading setup. The clay sample chamber was glued to a plexiglass tube and a plexiglass pedestal. The plexiglass tube was 10 mm thick and 150 mm high, with an inner diameter of 80 mm. Stainless steel circular hoops were used on the plexiglass tube to limit the deformation, which can obtain k0 conditions. The plexiglass pedestal was 20 mm thick with a diameter of 100 mm. The anode (79 mm diameter, MMO Ti mesh), anode filter paper, clay sample, cathode filter paper, cathode (79 mm diameter, stainless steel), and drainage groove were arranged from top to bottom in the clay sample chamber. The anode and the cathode were connected to a power supply (R2009) using wire belonging to the power supply system. There was a temperature measurement hole at a distance of 15 mm from the surface of the cathode. In the center of the plexiglass pedestal, a drainage hole was used to discharge water. The pressure loading setup included a pressure plate and weight. The pressure plate was made of plexiglass with an diameter of 79 mm and a thickness of 80 mm, which was used to apply pressure loading to the clay sample; the pressure loading was applied by static weights.

The data monitoring system carried out the monitoring of the current, settlement, temperature, and drainage volume. A current sensor (XIANMIAO) was connected to the power supply (R2009) and the anode using a wire in the circuit. A displacement sensor (MILANG) was utilized to monitor and record the settlement of the clay sample. A temperature sensor and a temperature collector (XIANMIAO_NTC) were used to monitor and record the temperature of the clay sample. The drainage volume was measured by a measuring cylinder.

### Experimental procedures

The experiment plan included electro-osmotic consolidation experiments and clay property measurement experiments, which are shown in Table 2. Both the duration and the voltage had three levels. The experimental procedures included the following steps:

1. Vaseline was smeared on the internal surface of the electro-osmotic consolidation cell to reduce the friction force between the pressure plate and the internal surface of the electro-osmotic consolidation cell.
2. A drainage groove, a cathode, and cathode filter paper were put on the plexiglass pedestal, successively. The slurry sample (60% water content) was poured into the electro-osmotic consolidation cell and then a vibration process was conducted to avoid bubbles inside slurry sample. Anode filter paper and an anode was put on the slurry sample, successively.
3. The pressure plate and a 25 kPa weight were applied to the anode for 72 h to conduct the consolidation process. After the settlement of the clay sample was stable, the power supply was turned on using the durations and voltages in Table 2. During the electro-osmotic consolidation experiments, the current, temperature, drainage volume, and settlement were monitored and recorded.
4. After the electro-osmotic consolidation experiments, the clay sample was taken out from the electro-osmotic consolidation cell. The diameter of the clay cross-section was measured using a Vernier caliper (GSD-03741B) at a normalized distance of 0, 0.2, 0.4, 0.6, 0.8, and 1 to the anode, corresponding to the boundaries of five equal parts. A Vernier caliper was fixed on the horizontal holder. When we measured the diameter of the clay cross-section, the measuring jaw was closed carefully over clay sample. The clay sample was divided into five equal measurement clay samples. The water content of the measurement clay samples was obtained based on the soil test standard SL237-1997. The remaining parts of the measurement clay samples were dried following the air-drying procedure.
5. The air-dried measurement clay samples were pulverized into clay powder. The clay powder was then mixed with distilled water (clay mass/distilled water mass = 1:5) to make slurry samples in centrifuge tubes. The centrifuge tubes were placed into a centrifugal machine (CT15RT) to make supernate samples. A pH meter (METTLER TOLEDO FE-28) and an ionometer (PXSJ-216F) were used to measure the pH value and the molar concentration of chloridion respectively, using the supernate samples.

**Table 2:** Experimental plan.

Experimental condition	Voltage (V)	Duration (h)
	6	48
	9	48
	12	48
	9	12
	9	24

## Results and discussion

The electro-osmotic consolidation included electrochemical processes (electrolysis and the transport of ion), temperature changes, and mechanical processes. The following sections explain the interrelationship between these three processes based on the experimental data and relative theories.

### Electrochemical processes

During electro-osmotic consolidation, ions are transported in the soil under the direct current. The transport of ions contains diffusion, electromigration, and electro-osmotic flux advection, which are shown in Eq. (1):

1 $$\begin{aligned} & J_{j}^{d} = D_{j} \tau n\nabla ( - c_{j} ) \\ & J_{j}^{m} = u_{j} \tau nc_{j} \nabla ( - E) \\ & J_{j}^{e} = c_{j} k_{e} \nabla ( - E) \\ \end{aligned}$$

where *J*_*j*_^*d*^ is the diffusive mass flux, *J*_*j*_^*m*^ is the migration mass flux, *J*_*j*_^*e*^ is the electro-osmotic mass flux, *D*_*j*_ is the diffusion coefficient, $\tau$ is the tortuosity factor, *n* is the porosity of the clay samples, *c*_*j*_ is the molar concentration, *u*_*j*_ is the ionic mobility, *E* is the electrical potential, and *k*_*e*_ is the electro-osmotic permeability coefficient. From previous literature^26,27^, the electromigration of *J*_*j*_^*m*^ is a major contributing component to the total mass flux. Therefore, the mass flux of H^+^ and OH^−^ increased with a rise in the duration and voltage [as per Eq. (1)].

H^+^ and O_2_ are generated on the anode surface and OH^−^ and H_2_ are generated on the cathode surface during an electrolysis reaction. The electric current is expended in electrolysis reaction^26^. Therefore, the amount of H^+^ at the anode and of OH^−^ at the cathode has a positive correlation with the duration and voltage of the experiment (the current increases with a rise in the voltage, which will be discussed in “Temperature change process” section). The specific reaction equation is shown in Eq. (2):

2 $$\begin{aligned} & 2H_{2} O - 4e^{ - } = O_{2} \uparrow \, + \, 4H^{ + } \quad \left( {{\text{anode}}} \right) \\ & 4H_{2} O + 4e^{ - } = \, 2H_{2} \uparrow \, + \, 4OH^{ - } \quad \left( {{\text{cathode}}} \right) \\ \end{aligned}$$

**Figure 3:**
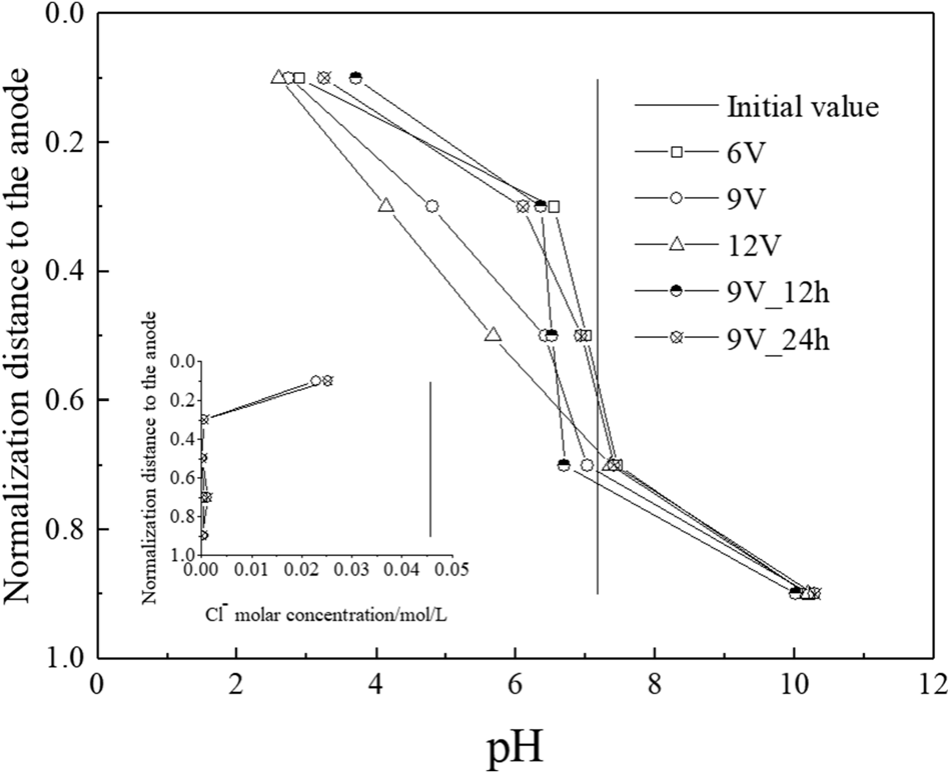
pH value and Molar concentration of Cl^−^ distribution.

Under the electromigration process, OH^−^ is transported from the surface of the cathode to the anode. The transport of H^+^ (transported from the surface of the anode to the cathode) can neutralize OH^−^ and can obstruct the transport of OH^−^. In addition, H^+^ is adsorbed by the surface of clay particles (negative charge) due to the buffering capacity of the clay (clay minerology: illite, kaolinite, and smectite)^27^. Therefore, the pH value increased from the anode to the cathode, as shown in Fig. 3. In addition, the pH value at a normalized distance of 0.1 to the anode decreased during electro-osmotic consolidation, and increased with a rise in voltage, which was mainly because the amount of H^+^ increased with a rise in the duration and voltage. In addition, existing Cl^−^ transported from the cathode to the anode under the electromigration process, and a part of Cl^−^ gathered near the anode^27^. Therefore, the molar concentration of Cl^−^ of the nearby anode was much higher than that of the nearby cathode, which was showed in the insert graph of Fig. 3. The insert graph of Fig. 3 shows that the molar concentration of Cl^−^ decreased with electro-osmotic going on. It is mainly because that the Cl^−^ lost electrons on the anode surface and generated chlorine, which reduced the molar concentration of Cl^−^ of the nearby anode.

### Temperature change process

The saturated clay sample consisted of clay particles and pore water. As per Eq. (3), the current value is determined by the external voltage and the apparent electrical resistance of the clay^28^. The apparent electrical resistance of the clay was determined by the apparent electrical resistivity of clay, as well as the clay sample’s geometrical shape.

3 $$I = \frac{{U_{m} }}{{R_{app} }} = \frac{{U_{m} }}{{\rho_{app} \frac{L}{S}}}$$

where *U*_*m*_ is the voltage, *R*_*app*_ is the apparent electrical resistance of the clay, $\rho_{app}$ is the apparent electrical resistivity of the clay. The apparent electrical resistivity of the saturated clay can be expressed by Eq. (4):

4 $$\rho_{app} = f(n,\rho_{w} ,T)_{{}}$$

The apparent electrical resistivity of the clay $\rho_{app}$ has a positive correlation with the electrical resistivity of the pore water $\rho_{w}$, and a negative correlation with porosity *n* and temperature *T*^28,29^.

**Figure 4:**
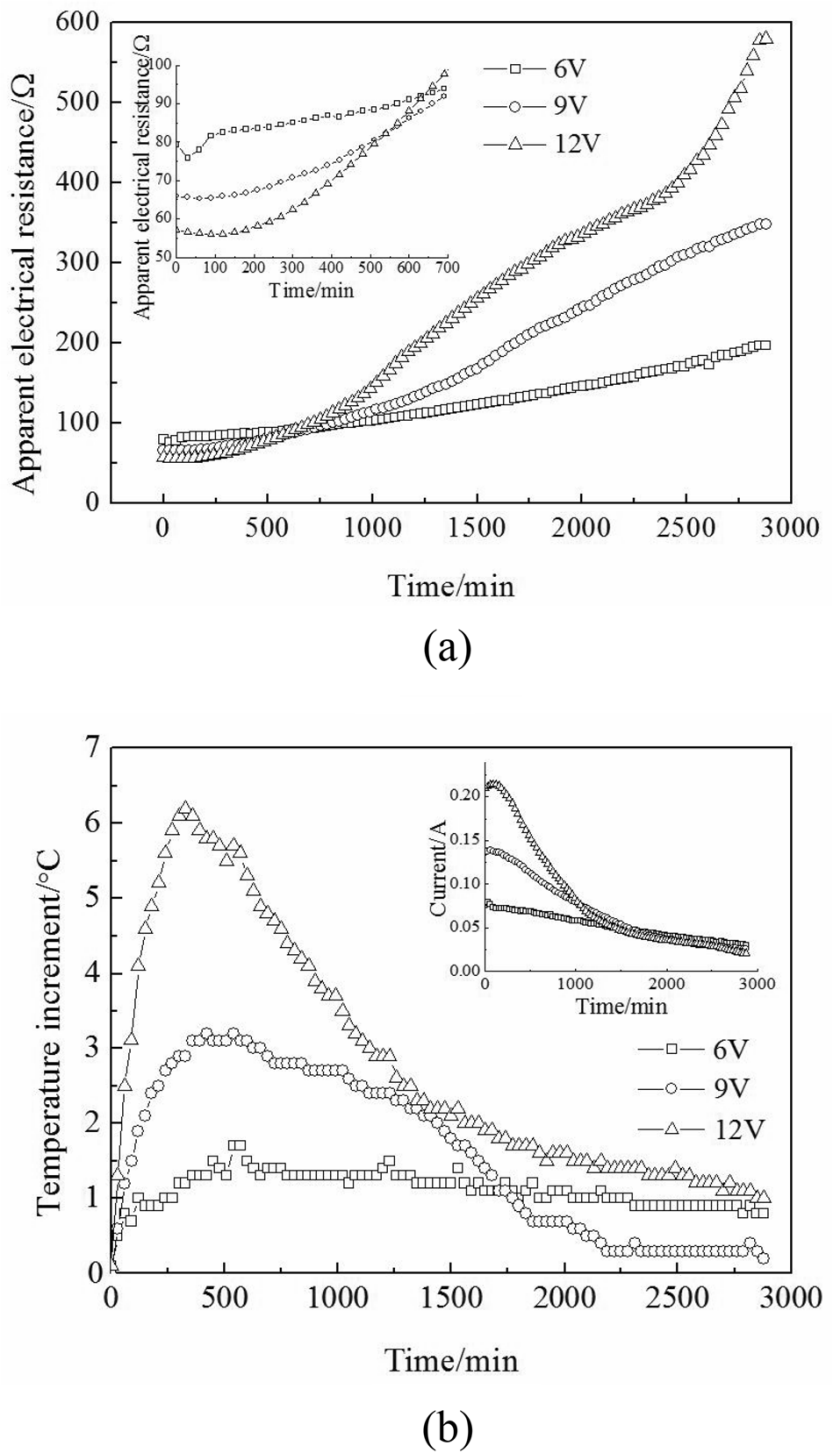
(**a**) Apparent clay electrical resistance; (**b**) temperature increment change under three voltages.

The curves of the apparent electrical resistance of the clay and the temperature change are shown in Fig. 4a, b. The apparent electrical resistance of the clay decreased in the initial 120 min of 12 V. Although the porosity *n* decreased during electro-osmotic consolidation^13^, which increased the apparent electrical resistivity of the clay. However, there were two main contributions that led to a greater reduction in the apparent electrical resistance of the clay than that of the increase in apparent electrical resistance (which came from the decrease in porosity *n*). The two main contributions were: On the one hand, pore water contained an amount of Na^+^ [molar conductivity 5.01 × 10^3^/(S m^2^ mol^−1^)] and Cl^−^ [molar conductivity 7.63 × 10^3^/(S m^2^ mol^−1^)] in the initial stage. The electrolysis reaction (discussed in “Electrochemical processes” section) produced H^+^ [molar conductivity 34.98 × 10^3^/(S m^2^ mol^−1^)] and OH^-^ [molar conductivity 19.8 × 10^3^/(S m^2^ mol^−1^)] during electro-osmotic consolidation, which decreased the apparent electrical resistance in this period. On the other hand, the Joule heating generated in the marine clay samples due to conveyance of the current increased the clay samples’ temperature (as shown in Fig. 4b). As per Eq. (4), the apparent electrical resistivity of the clay decreased with a rise in temperature.

As the pore water discharged from the cathode, the average water content decreased (which will be discussed in “Mechanical process” section), which increased the apparent electrical resistivity value of the clay. At the same time, the temperature decreased with a rise in the apparent electrical resistivity of the clay and reached a stable value at a later period of the electro-osmotic consolidation experiments, as shown in Fig. 4a, b.

As per Eq. (3), a high-voltage current is higher than that of a low-voltage current when the clay samples’ electrical resistance is the same. Based on Joule’s law, a high current corresponds to a high Joule heating. Therefore, the maximum temperature rise of 12 V was higher than that of 6 V, which is shown in Fig. 4b.

### Mechanical process

#### Electro-osmotic permeability coefficient and pore water pressure

**Figure 5:**
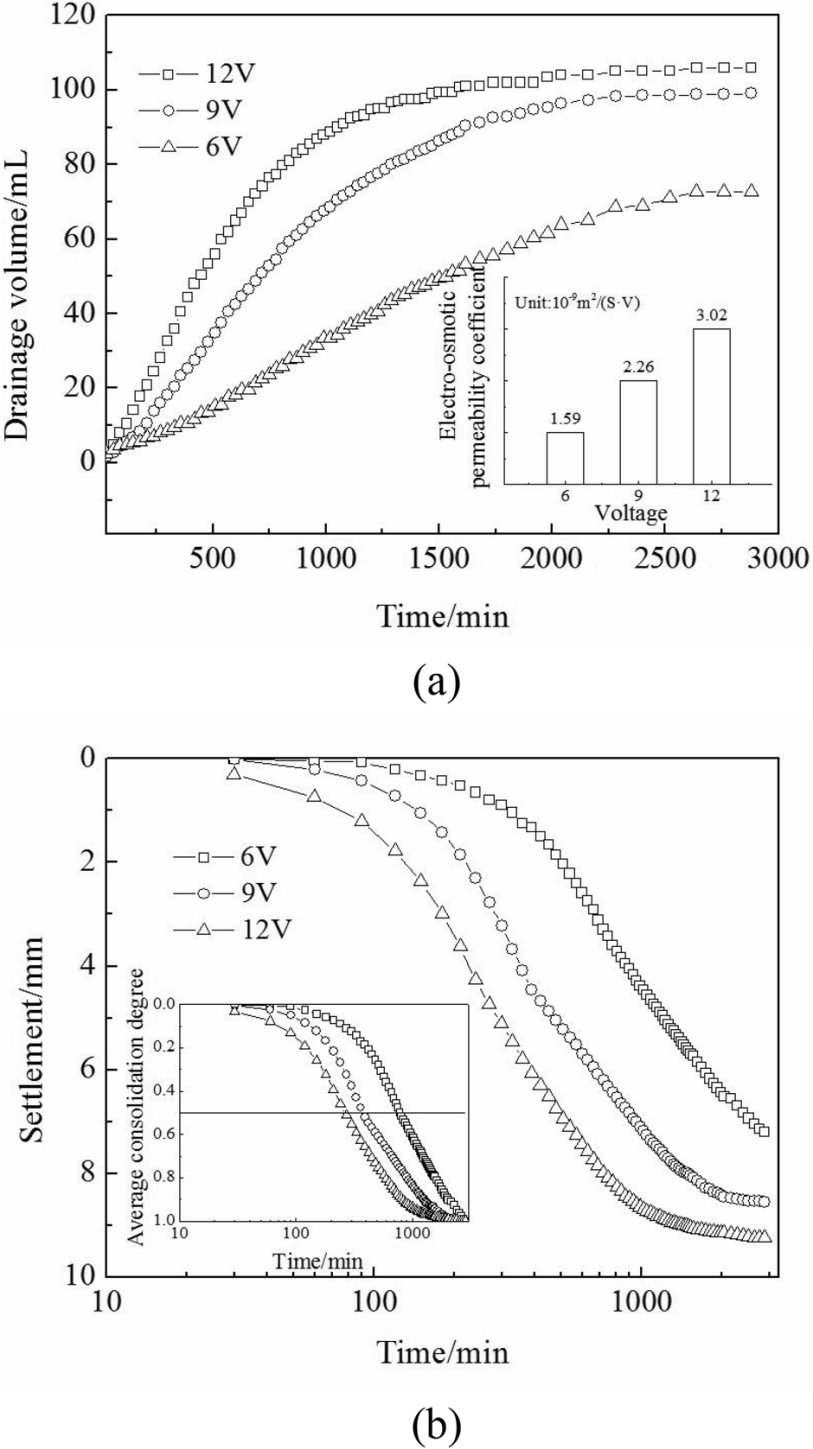
(**a**) Drainage volume; (**b**) settlement and average degree of electro-osmotic consolidation, changing over time under three voltages.

The electro-osmotic permeability coefficient *k*_*e*_ can be calculated by the drainage volume *Q* in Fig. 5a, the electrical potential gradient *i*_*e*_, the clay sample cross-section *A*, and the experiment time $\Delta t$.

5 $$k_{e} = \frac{Q}{{i_{e} A\Delta t}}$$

The *k*_*e*_ values (which are shown in the inset graph of Fig. 5a) increased with a rise in voltage in the initial 300 min, which was mainly because the temperature rise promoted a rise in the electro-osmotic permeability coefficient *k*_*e*_^30^.

Equation (6) is the expression of the consolidation coefficient, and Eq. (7) is the expression of negative pore water pressure *u*(*x*,*t*)^31^.

6 $$C_{v} = \frac{{k_{h} }}{{m_{v}\gamma_{w}}}$$

7 $$u\left( {x,t} \right) = - \frac{{k_{e} \gamma_{w} }}{{k_{h} }}\frac{{U_{m} x}}{L} + \frac{{2k_{e} \gamma_{w} U_{m} }}{{k_{h}\pi^{2} }}\sum\limits_{n = 0}^{\infty } {\frac{{\left( { - 1} \right)^{n} }}{{\left( {n + \frac{1}{2}} \right)^{2} }}} \cdot sin\frac{{\left( {n + \frac{1}{2}} \right)\pi x}}{L}\left[ {exp - \left( {n + \frac{1}{2}} \right)^{2} \pi^{2} T_{v} } \right]{\kern 1pt} {\kern 1pt} {\kern 1pt} {\kern 1pt} {\kern 1pt}$$

where *k*_*h*_ is the hydraulic permeability coefficient in the vertical direction, *x* is the distance to the cathode, *C*_*v*_ is the consolidation coefficient, and *m*_*v*_ is the coefficient of volume compressibility.

As per Eq. (7), the pore water pressure *u*(*x*, *t*) is a negative value and the absolute value of the pore water pressure *u*(*x*, *t*) increased during electro-osmotic consolidation. Based on the effective stress principle, the effective stress $\sigma^{^{\prime}}$ increases with the development of negative pore water pressure. The settlement of consolidation is caused by a rise in the effective stress, and was observed during electro-osmotic consolidation, which is shown in Fig. 5b. A high temperature increased the hydraulic permeability coefficient *k*_*h*_^32^. Therefore, a rise in voltage increased the consolidation coefficient *C*_*v*_^13^. *C*_*v*_ had a negative correlation with the 50% degree of consolidation time^4^, increasing with the rise in voltage (inset graph of Fig. 5b).

**Figure 6:**
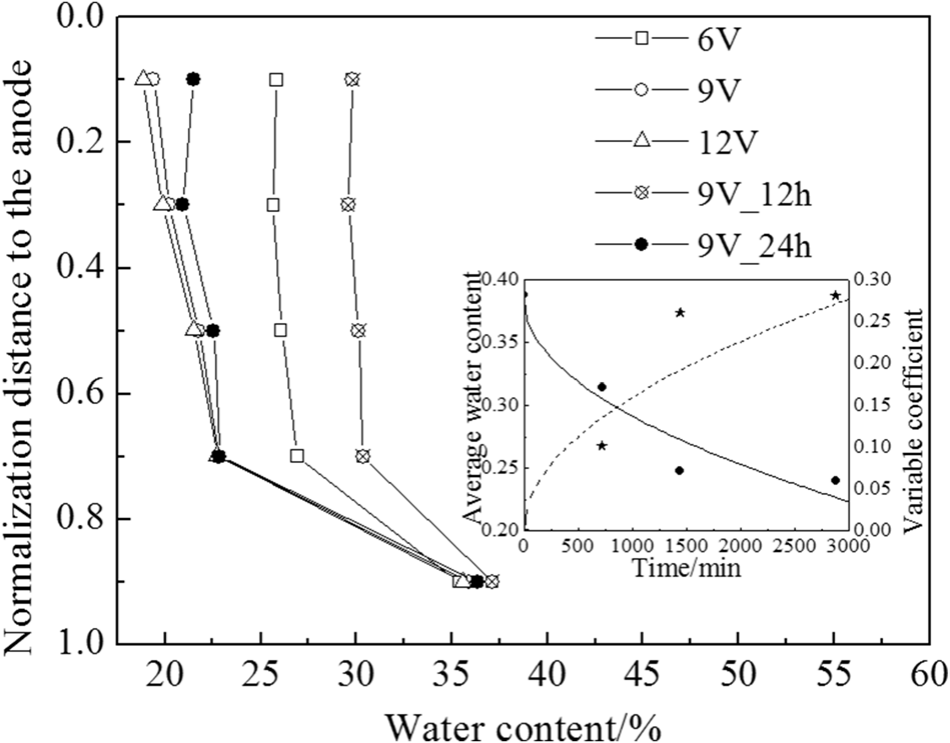
Water content distribution, average water content, and water content variable coefficient.

Correspondingly, the average water content reduced, as shown in the inset graph of Fig. 6. The absolute value of the negative pore water pressure and the effective stress $\sigma^{^{\prime}}$ increased from the cathode to the anode, which had a positive correlation with voltage. Therefore, the water content decreased from the cathode to the anode and decreased with the rise in voltage during electro-osmotic consolidation, which is shown in Fig. 6. Furthermore, the settlement of 12 V was higher than that of 9 V and 6 V after 48 h electro-osmotic consolidation.

The variable coefficient *cov* was used to measure the water content discreteness among the clay samples:

8 $${\text{cov}} = \frac{{\sigma_{water\_content} }}{{\mu_{water\_content} }}$$

where $\mu_{water\_content}$ is the mean value of the water content, and $\sigma_{water\_content}$ is the mean square error of the water content. The water content otherness in different positions increased with a rise in the variable coefficient. In the inset graph of Fig. 6, the water content variable coefficient *cov* increased during electro-osmotic consolidation. As can be seen from Eq. (7) and the previous literature^14^, the absolute value of the negative pore water pressure *u*(*x,t*) otherness increased during electro-osmotic consolidation. Based on the effective stress principle, the effective stress otherness and water content otherness increased, correspondingly.

#### Horizontal shrinkage

During electro-osmotic consolidation, the negative pore water pressure *u*(*x*, *t*) induced the vertical and horizontal stress increment $\Delta \sigma_{eo} (x,t)$ in the clay samples. The critical stress condition of horizontal shrinkage can be expressed by Eq. (9), which was modified from^33^:

9 $$\Delta \sigma_{eo} (x,t) = \left| {u(x,t)} \right| > \frac{{k_{0} \cdot \sigma_{v0}^{,} }}{{1 - k_{0} }}$$

10 $$k_{0} = \left( {{1 - }\sin \phi^{^{\prime}} } \right) \cdot \left( {{\text{OCR}}} \right)^{{\sin \phi^{^{\prime}} }}$$

where *k*_*0*_ is the earth pressure coefficient, and $\sigma_{v0}^{^{\prime}}$ is the initial effective stress, which is equal to the initial consolidation pressure on the clay samples. *k*_*0*_ can be described by the stress history *OCR* and the effective internal friction angle $\varphi^{^{\prime}}$. When the horizontal stress increment $\Delta \sigma_{e0} {\text{(x,t) > 1}}{.8}\sigma_{v0}^{^{\prime}}$ [by putting the parameter $\varphi^{^{\prime}}$ = 20.9° into Eqs. (9) and (10)], horizontal shrinkage of the clay samples occurred. The horizontal shrinkage value can be calculated by Eq. (11):

11 $$D_{shrinkage} = D_{initial} {-} \, D_{treatment}$$

where *D*_*shrinkage*_ is the horizontal shrinkage value, *D*_*initial*_ is the initial diameter of the clay, and *D*_*treatment*_ is the diameter of a clay sample cross-section after electro-osmotic consolidation, which was measured by a caliper.

**Figure 7:**
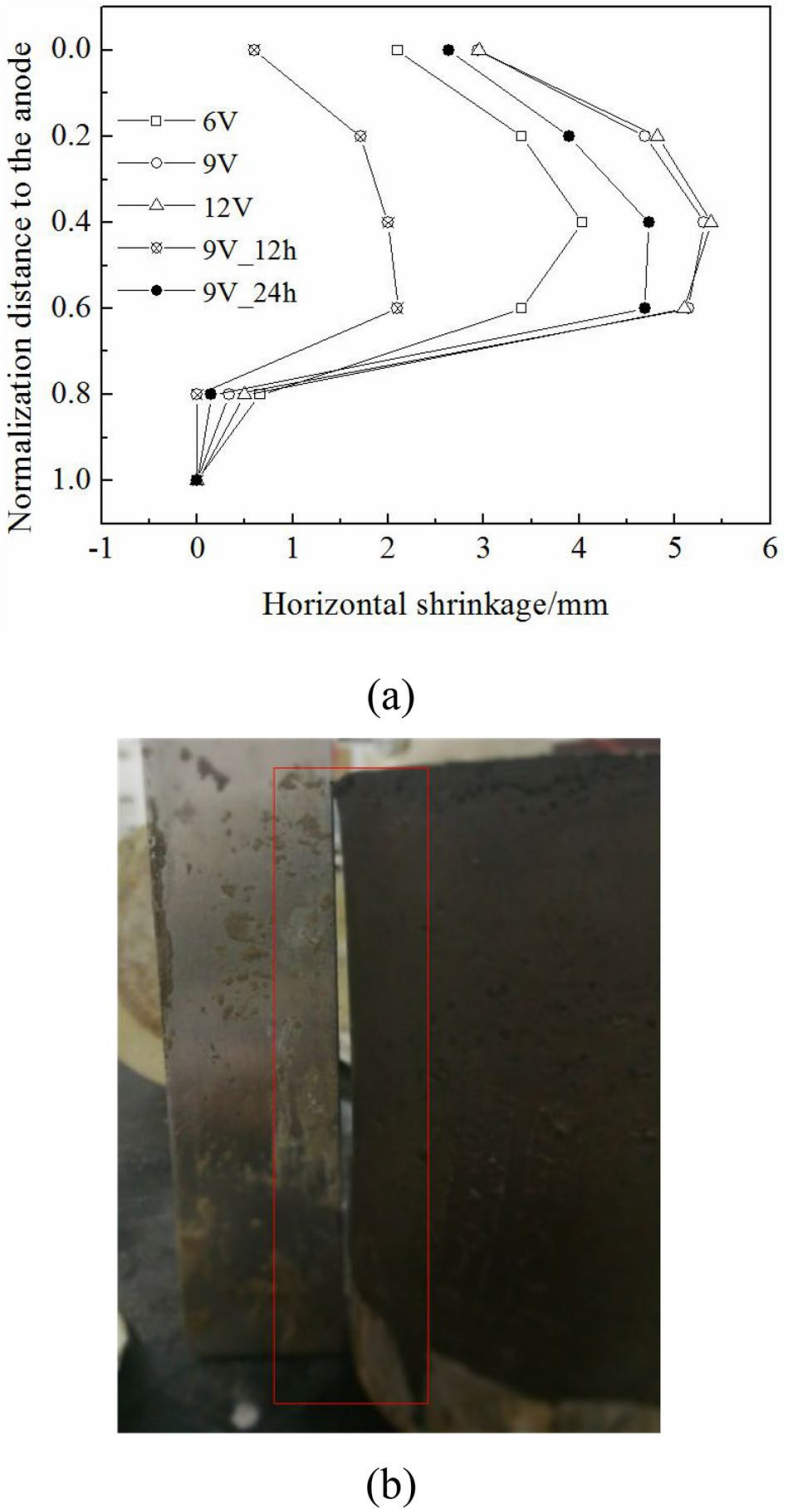
(**a**) Horizontal shrinkage of the clay samples after electro-osmotic consolidation; (**b**) schematic photo of the horizontal shrinkage.

Figure 7a shows that the horizontal shrinkage increased during the experiment, which was mainly because the horizontal stress increment $\Delta \sigma_{eo} (x,t)$ increased. As per Eqs. (9) and (7), the horizontal stress increment $\Delta \sigma_{eo} (x,t)$ increased from the cathode to the anode. In theory, the horizontal shrinkage value should increase from the cathode to the anode. However, anode–clay contact created a frictional force, which reduced the horizontal shrinkage value at the anode–clay contact site. Thus, maximum horizontal shrinkage did not appear at the anode–clay contact site. In addition, the horizontal shrinkage value was almost zero near the cathode (horizontal shrinkage schematic photo is shown in Fig. 7b). This was because the horizontal stress increment $\Delta \sigma_{eo} (x,t)$ in this area was lower than that of the critical stress value. As per Eqs. (7) and (9), the horizontal stress increment $\Delta \sigma_{eo} (x,t)$ was proportional to the external voltage value *U*_*m*_. Furthermore, horizontal shrinkage increased with a rise in the external voltage *U*_*m*_.

#### Volume differences

In the preloading consolidation experiment using an oedometer, no horizontal shrinkage occurred and bubbles were generated in the clay samples. Karunaratne and Chew et al.^3,34^ observed that the settlement of the soil foundation was not obvious after electro-osmosis consolidation; however, an increase in the drainage volume of the soil foundation was obvious during electro-osmotic consolidation, which was mainly because of the existence of a “volume difference” . During the electro-osmotic consolidation of marine clay: (1) Bubbles were produced by the electrolysis reaction^35^ and moved into the marine clay, resulting in an “unsaturated” status of the marine clay^36^; (2) horizontal shrinkage accompanied settlement during electro-osmotic consolidation. Due to these two reasons, the drainage volume was much higher than the change in vertical settlement volume *V*_*vertical*_. The volume difference of electro-osmotic consolidation can be expressed by Eq. (12):

12 $$\Delta V = V_{all} - V_{vertical} = V_{shrinkage} + V_{air}$$

where *V*_*all*_ is the drainage volume of electro-osmotic consolidation; *V*_*vertical*_ is the change in vertical settlement volume; *V*_*shrinkage*_ is the change in horizontal shrinkage volume; and *V*_*air*_ is the bubble volume in the clay samples.

**Figure 8:**
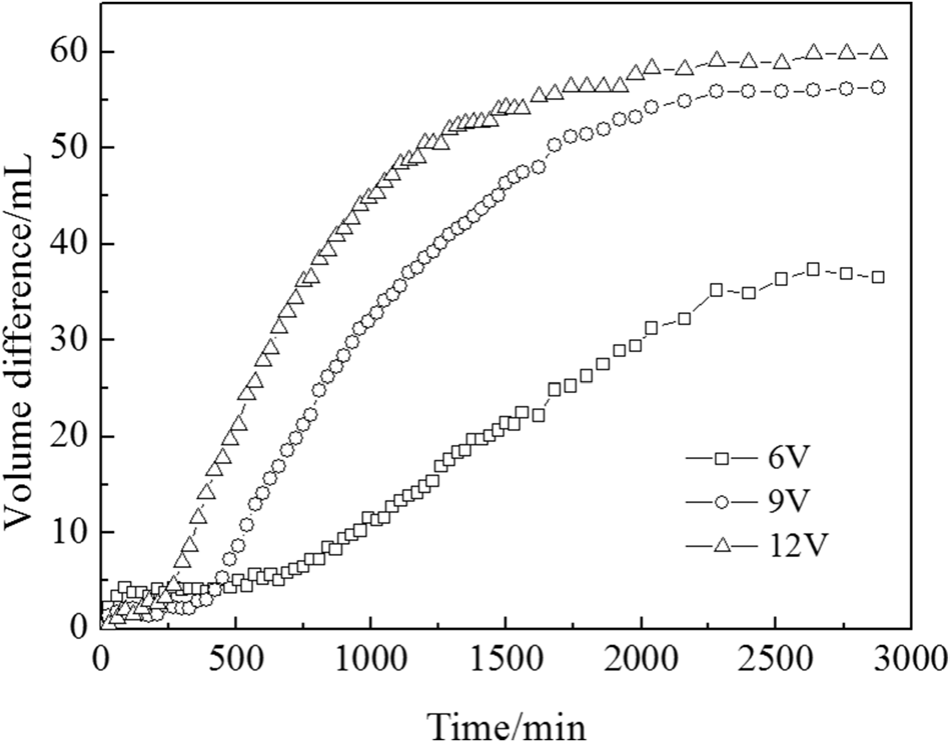
The difference in volume, changing over time under different durations and voltages.

Horizontal shrinkage and bubbles in the marine clay increased during electro-osmotic consolidation, which was discussed in “Electrochemical processes” and “Horizontal shrinkage” sections. As a result, the volume difference $\Delta V$ increased with time, as shown in Fig. 8. In addition, the horizontal shrinkage and the amount of bubbles (i.e., the electrolysis reaction) increased with a rise in voltage *U*_*m*_, as shown in Fig. 7a and in the discussion of “Horizontal shrinkage” section. Therefore, the higher the voltage, the higher the volume difference.

### Coupling analysis of the electro-osmotic consolidation process

**Figure 9:**
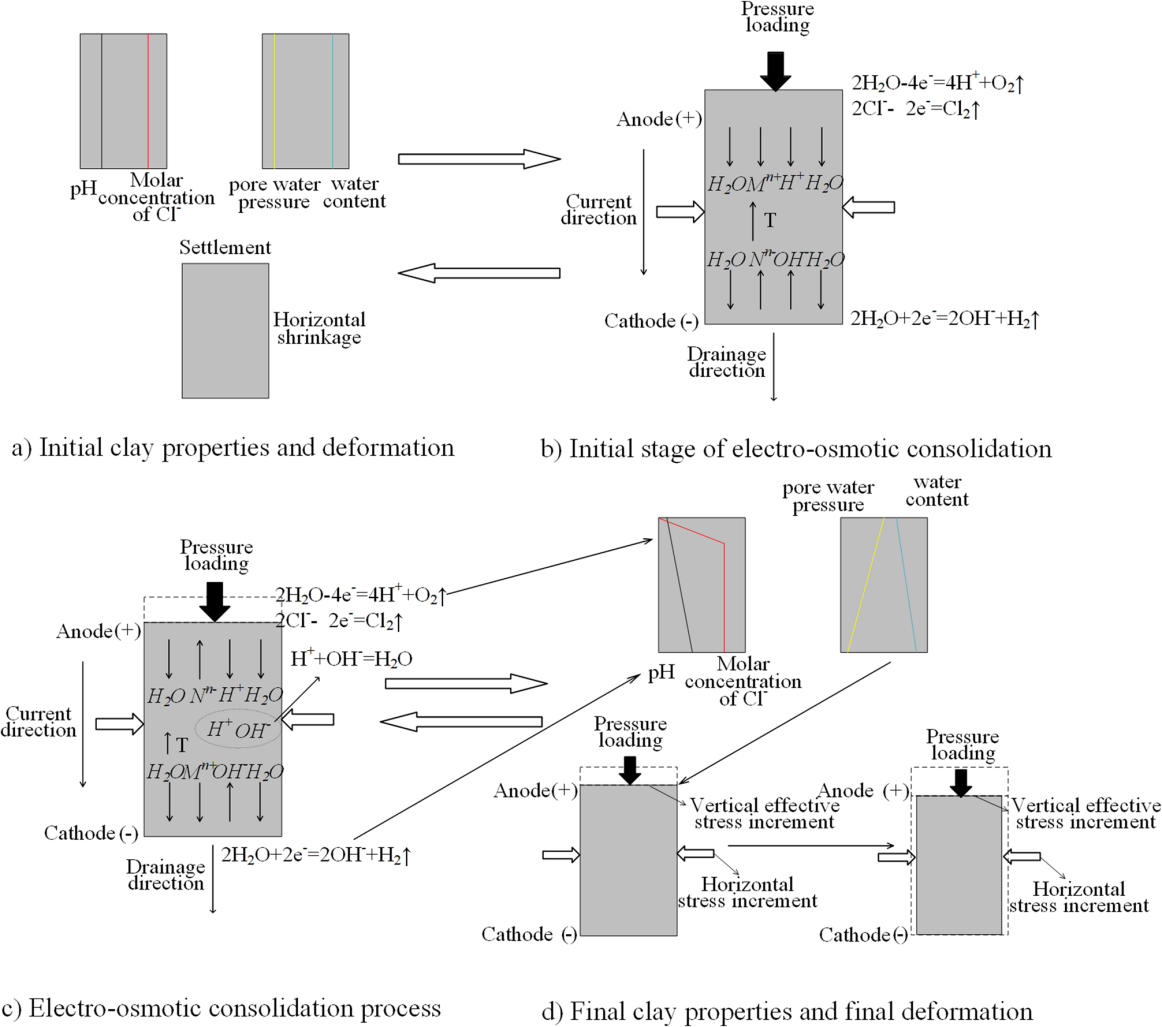
Diagram of the combination of electrochemical–temperature–mechanical processes during electro-osmotic consolidation.

In Fig. 9a, we assumed that the initial clay properties were uniform. As shown in Fig. 9b, when direct current was applied to the marine clay samples, the pore water moved from the anode to the cathode, and then discharged from the cathode. As a result, the average water content reduced during the electro-osmotic consolidation. At the same time, an absolute value of the negative pore water pressure *u*(*x*, *t*) developed and increased from the cathode to the anode, which induced vertical effective stress and increased the horizontal stress increment $\Delta \sigma_{eo} (x,t)$. Firstly, vertical settlement occurred in the marine clay samples, but horizontal shrinkage was not observed in the initial stage. Secondly, the horizontal stress increment $\Delta \sigma_{eo} (x,t)$ was higher than the critical stress condition, and horizontal shrinkage and volume difference were observed, which is shown in Fig. 9d. Vertical settlement and horizontal shrinkage reduced the porosity of the clay samples.

During electro-osmotic consolidation, an electrolysis reaction occurred on the surface of the electrodes, which is shown in Fig. 9b, c. H^+^ (anode product) was transported from the anode to the cathode, while OH^-^ had the opposite transport direction. During the transport of ions, H^+^ and OH^-^ met in the middle section of the marine clay samples, which initiated a neutralization reaction. Due to the high water content and the conductivity in the clay near the cathode, the electrical potential was much lower than the ideal value, which reduced the effect of the reduction in water content near the cathode. In addition to H^+^ and OH^−^, the surface of the electrode produced bubbles, and the bubbles moved into the marine clay samples, which is another reason for the volume difference.

A temperature rise was observed in this research, which was caused by Joule heating. The temperature increased with a rise in the current, but decreased with a reduction in the porosity of the clay samples. It is beneficial for the mass flux of ions to increase, because effective ionic mobility has a positive relationship with temperature. Moreover, a rise in temperature promoted the electro-osmotic consolidation effect and the consolidation coefficient, which increased the effect of the reduction in water content and the consolidation coefficient. However, the temperature rise induced an increase in the energy coefficient.

## Conclusions and prospects

To explore the mechanism of electro-osmotic consolidation, this paper conducted a series of experiments under different durations and voltages. Based on the experimental data and the relevant theories, the following conclusions were drawn:

1. The amount and mass flux of H^+^ and OH^−^ increased during electro-osmotic consolidation and with a rise in voltage. For example, pH increased from 7.17 to 10.23 near the cathode and decreased from 7.17 to 2.74 near the anode during 9 V electro-osmotic consolidation.
2. Joule heating increased the temperature of the marine clay during electro-osmotic consolidation. The temperature increment increased to 6.2 °C during 12 V electro-osmotic consolidation. In addition, the rise in temperature promoted the mechanical process of electro-osmotic consolidation.
3. Horizontal shrinkage has a positive relationship with the duration of electro-osmotic consolidation, voltage, and *k*_*e*_/*k*_*h*_. Therefore, the volume difference increased with the duration and a rise in voltage, correspondingly. In addition, the volume difference included the contribution of bubbles. The volume difference reached 59.8 mL during 12 V electro-osmotic consolidation.
4. An analytical solution or a numerical simulation should be provided to describe the combination of electrochemical–temperature–mechanical processes, which will be treated as the next research topic.

## Supplementary information

Supplementary Information.

## Data Availability

The datasets generated and/or analyzed during the current study are available from the corresponding author on reasonable request.
